# Effect of in situ CO_2_ mixing of cement paste on the leachability of hexavalent chromium (Cr(VI))

**DOI:** 10.1007/s11356-024-34582-2

**Published:** 2024-08-08

**Authors:** Kian Cho, Won Kyung Kim, Juhyuk Moon, Daniel Cha, Junboum Park

**Affiliations:** 1 Department of Civil and Environmental Engineering, Seoul National University, Seoul, South Korea 08826; 2 Department of Civil and Environmental Engineering, University of Delaware, Delaware, 19716 USA

**Keywords:** Carbon Capture and Utilisation (CCU) technology, Ordinary Portland cement, Leaching test, Hexavalent chromium, Monocarboaluminate, Chromate hydrate, Microstructural analysis

## Abstract

In situ CO_2_ mixing technology is a potential technology for permanently sequestering CO_2_ during concrete manufacturing processes. Although it has been approved as a promising carbon capture and utilisation (CCU) method, its effect on the leachability of heavy metals from cementitious compounds has not yet been studied. This study focuses on the effect of in situ CO_2_ mixing of cement paste on the leaching of hexavalent chromium (Cr(VI)). The tank leaching test of the CO_2_ mixing cement specimen resulted in a Cr(VI) cumulative leaching of 0.614 mg/m^2^ in 28 d, which is ten times lower than that of the control mixing specimens. The results in thermogravimetric analysis indicated that a relatively significant amount of CrO_4_^2−^ is immobilised as CaCrO_4_ during the CO_2_-mixing, and a higher Cr–O extension is observed in the Fourier transform infrared spectra. Furthermore, a portion of the monocarboaluminate is inferred from microstructural analyses to incorporate CrO_4_^2−^ ions. These results demonstrate that in situ CO_2_ mixing is beneficial not only in reducing CO_2_ emissions, but also in controlling the leaching of toxic substances.

## Introduction

Chromium is one of the 25 most widespread elements in the Earth’s crust (Emsley 2011). It exists in various forms, however hexavalent chromium (Cr(VI)) is significantly toxic and highly carcinogenic (Mondal et al. 2021; Saha et al. 2022). Since 1990, the International Agency for Research on Cancer (IARC 2012) has classified Cr(VI) as a group 1 carcinogen, indicating its confirmed ability to cause cancer in humans. Cr(VI) is used in various industrial processes, including medicine, catalysis, fuel production, leather tanning, electroplating, and pigment manufacturing (O'Neil 2013; Saha et al. 2011). Despite its utility, Cr(VI) poses severe health risks, such as damage to nasal epithelia, skin ulcers, known as 'chrome holes', and lung cancer when inhaled (Wu et al. 2020b). It can penetrate cells, causing DNA damage and oxidative stress, which contributes to its carcinogenic properties (Costa and Klein 2006). Environmentally, Cr(VI) contamination in water sources poses significant risks, necessitating strict regulatory control to mitigate exposure and protect both human health and the ecosystem. Recently chromium removal technologies highlighted a diverse range of effective methods (Mukherjee et al. 2013) including bioremediation (Costa and Klein 2006; Saha and Orvig 2010; Saha and Saha 2014), ion exchange (Rengaraj et al. 2001), membrane filtration (Ho and Poddar 2001), adsorption (Pakade et al. 2019), and electrochemical techniques (Liu et al. 2011).

Hexavalent chromium (Cr(VI)) has been widely recognised as a heavy metal that is eluted from cement-based materials (Eštoková et al. 2012; He et al. 2023). When Cr(VI) comes into direct contact with the human skin, it can trigger hypersensitivity reactions and allergic dermatitis, particularly for construction workers who work with cement or fresh concrete (Frías and Sánchez de Rojas 2002;Scrivener et al. 2016). Therefore, the presence of Cr(VI) compounds in cement-based materials may pose severe environmental and public health challenges owing to their solubility and potential leaching from cementitious materials, such as water tanks, pipes, or cementation of soils. The leaching of Cr(VI) from cementitious materials is caused by the dissolution of chromate (CrO_4_^2−^) in the pore solution of the cement paste, which is an alkaline environment. Immobilisation of CrO_4_^2−^ is assumed to occur because of ion exchange in calcium aluminate hydrates (Perkins and Palmer 2000, 2001; Pöllmann and Auer 2012). The chromium compounds such as chromate-ettringite (Ca_6_·Al_2_·(OH)_12_·(CrO_4_)_3_·26H_2_O) or monochromate (Ca_4_·Al_2_· (OH)_12_·CrO_4_·6H_2_O) (Leisinger et al. 2012; Takahashi et al. 2003) can be generated by incorporating CrO_4_^2−^.

The in situ CO_2_ mixing technology of cement paste is attracting global interest, as the cement industry accounts for a large portion of global CO_2_ emissions (United Nations Environment Programme 2020). The main idea of carbon capture and storage (CCS) or carbon capture and utilisation (CCU) technology is to collect CO_2_ at CO_2_-emitting processes, such as cement manufacturing, and inject and sequester it into stable sites or materials (Pacala and Socolow 2004; Sanna et al. 2014). CCS or CCU is feasible in the cement industry under favourable environmental conditions, such as abundant calcium ions (Ca^2+^) and high pH in the cement paste, as stated in Eq. 1 and 2.

1 $${\text{C}}_{3}\text{S }+3{\text{H}}_{2}\text{O}\leftrightarrow 3{\text{Ca}}^{2+}+{\text{SiO}}_{2}+6{\text{OH}}^{-}$$

2 $${\text{C}}_{2}\text{S }+2{\text{H}}_{2}\text{O}\leftrightarrow 2{\text{Ca}}^{2+}+{\text{SiO}}_{2}+4{\text{OH}}^{-},$$

where C_3_S is tricalcium silicate, and C_2_S is dicalcium silicate. In situ CO_2_ mixing technology or carbon curing of concrete is a mineral carbonation technique in which CO_2_ reacts with Ca^2+^ ions to deposit calcium carbonate (CaCO_3_) (Li et al. 2019). As CO_2_ gas dissolves in the cement slurry, it forms CO_3_^2−^, and CaCO_3_ precipitates via the combination of Ca^2+^ and CO_3_^2−^, as shown in Eq. 3.

3 $${\text{Ca}}^{2+}+{\text{CO}}_{3}^{2-}\leftrightarrow {\text{CaCO}}_{3}$$

The CO_2_ mixing that induces cement carbonation has been reported to have some impact on the mechanical and durability properties of cement (El-Hassan and Shao 2015; Li et al. 2019). However, its effect on the leachability of heavy metals has not yet been studied. Therefore, this study aims to evaluate the leachability of Cr(VI) from ordinary Portland cement (OPC) when the cement paste is partially carbonated via in situ CO_2_ mixing. The specimens mixed under laboratory conditions (i.e. air mixing) and high CO_2_ concentration conditions (i.e. CO_2_ mixing) are compared in terms of the leachability of Cr(VI) through a tank leaching test. To investigate the transformation of cement hydrates, the following microstructural analyses are applied: X-ray diffraction analysis (XRD), thermogravimetric analysis (TGA), and Fourier transform infrared (FTIR). For these microstructural analyses, a Cr(VI) solution was added instead of water to observe the effect of CO_2_ mixing on the Cr(VI) immobilisation mechanism in the cement paste.

## Materials and methods

### Specimen preparation

Commercial OPC (Hannil, South Korea) was used in this study. Table 1 presents the chemical composition of the OPC, as measured by the X-ray fluorescence (XRF) analysis. Table 2 lists detailed descriptions of each experimental condition. The cement paste was mixed in either a laboratory environment (air mixing) or a glove box filled with a high concentration of CO_2_ gas (CO_2_ mixing). The water-to-cement (w/c) ratio was 0.5, comprising 200 g of cement and 100 g of a water-or Cr(VI)-saturated mixing solution. The Cr(VI)-saturated mixing solution was prepared by dissolving 5 g of potassium dichromate (K_2_Cr_2_O_7_, DUKSAN Company, South Korea) in 100 mL of deionised water (i.e., 17.67 g/L of Cr(VI) solution). After mixing with cement under each experimental condition, the paste was moulded in a cylinder (2.5 cm I.D. × 2.5 cm H), sealed with plastic wrap, and tied with a rubber band. The specimens were cured in an environmental chamber at a constant temperature of 20 °C and humidity of 60%.

**Table 1:** Chemical composition of OPC by XRF

Name	SiO_2_	Al_2_O_3_	TiO_2_	Fe_2_O_3_*	MgO	CaO	Na_2_O	K_2_O	MnO	P_2_O_5_	LOI**	Total
%	19.93	4.61	0.21	3.12	2.74	63.21	0.09	0.93	0.09	0.21	3.02	98.2

*Fe_2_O_3_: Total Fe, **LOI: Loss of ignition

**Table 2:** Material designs in the experiment

Name	Mixing	Cr(VI) addition (mg/L)	Water (g)	Cement (g)
air_cr0	Air	0	100	200
CO_2__cr0	CO_2_			
air_cr5	Air	50		
CO_2__cr5	CO_2_			

### CO_2_ mixing of cement paste

**Fig. 1:**
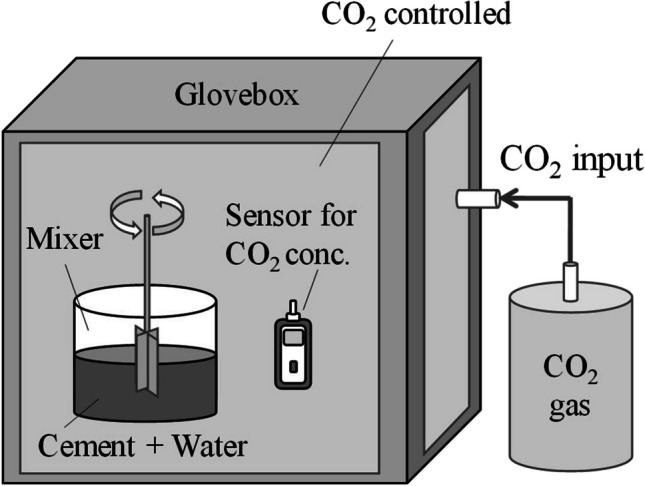
Schematic illustration of CO_2_-mixing method

To implement in situ CO_2_ mixing of the cement paste, a glove box was used to mix the cement paste under a specific CO_2_ concentration (Fig. 1). The beaker containing 100 mL of water or Cr(VI) solution, OPC, the mixer, and the CO_2_ sensor (SKY2000, Shenzhen YuanTe Technology Co., Ltd., Shenzhen, China) were placed inside the glove box. Subsequently, the glove box was filled with CO_2_ (Purity: 99.999%, KS Tech Co., Ltd., Anseong, South Korea) to a concentration of approximately 10 vol%. Moreover, the water and cement were mixed in the glove box for 30 min. The CO_2_ concentration in the glove box was recorded every 5 min. A condition without mixing was also tested to evaluate the CO_2_ reduction under unmixed conditions.

### Tank leaching test

**Fig. 2:**
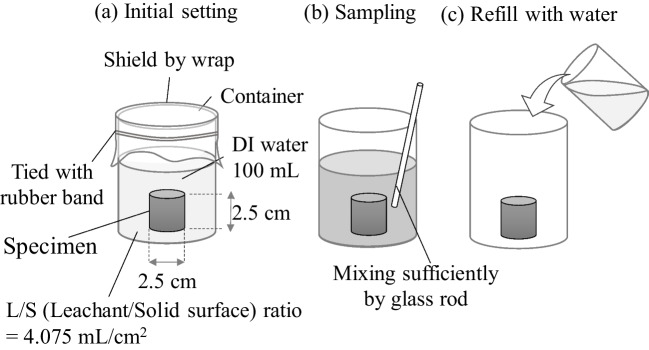
Schematic illustration of the procedure in the tank leaching test

Figure 2 illustrates the tank leaching test. The test was performed at an ambient temperature of 22 ± 1 °C and a relative humidity of ~ 60%. The ratio of the volume of the leachate to the surface area of the specimen (L/S ratio) was approximately 4.07 mL/cm^2^. The specimen was placed in a 200-mL beaker containing 100 mL of deionised water as leachate. To avoid evaporation of the leachate, the beaker was tightly sealed with plastic wrap. After specific leaching periods of 1, 2, 4, 8, 16, and 28 d, the leachate was stirred gently, and leachate samples were collected. The leaching tests were conducted on five samples for each mixing method. Subsequently, the leachate was discarded, and the beaker was refilled with deionised water.

The collected leachate was analysed for Cr(VI) concentrations to quantify the Cr(VI) leaching from the cement specimens according to the European Standard method (EN196-10:2016 2016). Briefly, 1,5 di-phenyl-carbohydrazide (C_13_H_14_N_4_O) was used to form a 1,5 di-phenyl-carbohydrazide-Cr(VI) complex in a dilute acid solution (0.04 M HCl). The resulting Cr(VI) complexes were analysed using a UV–visible spectrometer (Cary 3500 UV–Vis Multicell, Agilent Technologies, Inc., USA) at a wavelength of 540 nm.

The Environmental Protection Agency (EPA1315 2013) proposed mass transfer rates of inorganic compounds in cement under diffusion-controlled release conditions as a function of leaching time. The observed diffusivity (*D*_obs_) was determined by analysing the leaching test results, as shown in Eq. 4.

4 $${D}_{\text{iobs}}=\pi {\left[\frac{{M}_{\text{ti}}}{2\rho {C}_{0}\left(\sqrt{{t}_{\text{i}}}-\sqrt{{t}_{\text{i}-1}}\right)}\right]}^{2}$$

where* D*_iobs_ is the observed diffusion coefficient for each interval (cm^2^/day), *M*_ti_ is the mass released during the leaching interval, i (mg/m^2^), *t*_i_ is the cumulative contact time at the end of the current leaching interval, i (s), *t*_i-1_ is the cumulative contact time at the end of the previous leaching interval, i-1 (s), $\rho$ is the density of the material (g/cm^3^), and *C*_0_ is the initial leachable content, that is, the available release potential (mg/kg). The *C*_0_ of Cr(VI) from OPC was 3.495 mg/kg (EN196-10:2016 2016).

### Microstructural analysis of cement paste

To prepare for microstructural analyses, a hydration stoppage was applied to preserve the samples and allow the analysis of various material properties at the same hydration stage (Snellings et al. 2018; Zhang and Scherer 2011). After pulverising, the specimens were soaked in a sufficient amount of isopropyl alcohol for 30 min to remove the free water trapped in the pores of the cement structure. The isopropyl alcohol was trimmed by vacuum pumping on filter paper (No. 2, with a pore size of 5$\mu$m). Subsequently, the sample was placed in a thermostatic oven maintained at 40 °C for approximately 5 min and soaked in ethyl ether for 30 min (i.e., expelling the isopropyl alcohol). Afterwards, the sample powder was trimmed again and dried in a thermostatic oven maintained at 40 °C for 40 min.

In this study, a TGA) was performed (SDT Q600, TA Instruments, USA). In the TGA, approximately 25–35 mg of the material was placed on the plate to avoid variations in the measurement. The ramps were 1.00 °C/min to 30 °C and 10.00 °C/min to 1000 °C. To observe the Cr(VI)-containing hydrates in the cement paste, additional measurements were performed with 1.00 °C/min to 20 °C and 10.00 °C/min to 1300 °C for 28-day cured samples. Moreover, an X-ray diffraction (XRD) analysis was performed (Bruker Co. Ltd., Germany). The initial setting of analysis was as follows: Cu-Kα line with a wavelength (λ) of 1.5418 Å, 2*θ* of 5–60°, the scanning rate of 2 min/°, and a step of 0.02°. FTIR spectroscopy was performed (TENSOR27, Bruker Co. Ltd., Germany). The spectral range was 400–4,000 cm^−1^, whereas the resolution was better than 0.4 cm^−1^ (apodised function), and the high-sensitivity DLATGS detector was used.

## Results and discussion

### Laboratory scale of in situ CO_2_ mixing

**Fig. 3:**
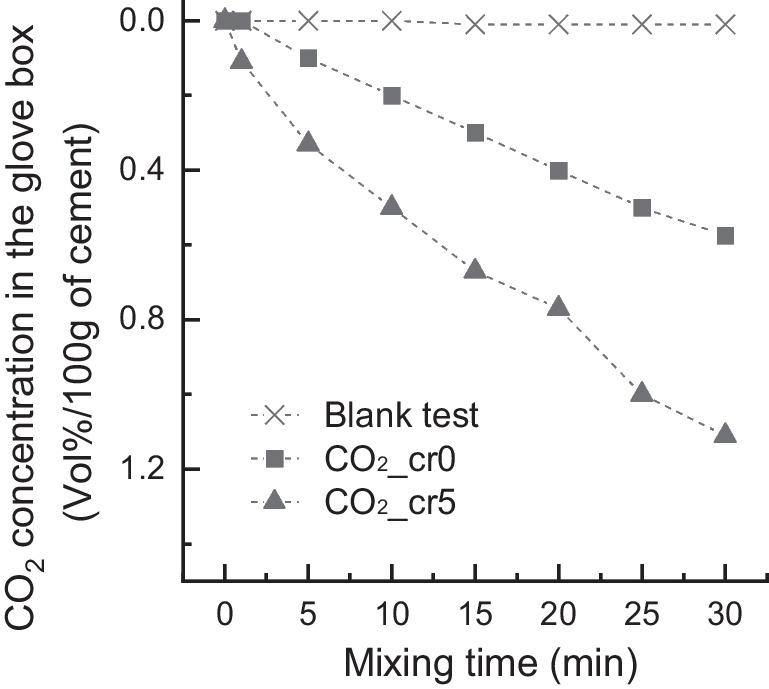
Reduction of CO_2_ concentration in the glove box during the mixing of 200 g cement

Figure 3 shows the concentrations of CO_2_ in the glove box during the mixing of 300 g of cement paste. The results showed that the experimental conditions of the glove box did not have a significant effect on the change in CO_2_ concentration because CO_2_ reduction was not observed in the unmixed beaker containing OPC, as implied in the blank test. Almost 0.6 vol% of CO_2_ decreased in the glove box for 30 min when the OPC paste was prepared with water (CO_2__cr0). Moreover, approximately 1.1 vol% of CO_2_ concentration decreased more rapidly during the same mixing period with the Cr(VI) addition of 50 mg/L to the mixing solution (CO_2__cr5). These results suggest that mixing the OPC cement paste under abundant CO_2_ conditions facilitates the absorption of CO_2_ by the cement paste. CO_2_ gas can react with Ca^2+^ dissolved in cement paste to form calcium carbonate (CaCO_3_) precipitates, according to Eq. 3. The greater absorption of CO_2_ during the mixing of the Cr(VI)-containing OPC paste may be attributed to the acidic pH of the CrO_4_^2−^ solution (O'Neil 2013). The dissolution of CO_2_ increased at a lower pH owing to the formation of carbonic acid (H_2_CO_3_).

### Tank leaching test

A tank leaching test was conducted to compare Cr(VI) leaching from air and CO_2_ mixed specimens. Five specimens of each mixing condition were subjected to the leaching tests. Table 3 lists the mass and dimensions of the 28-day cured specimens at the start of the leaching test.

**Fig. 4:**
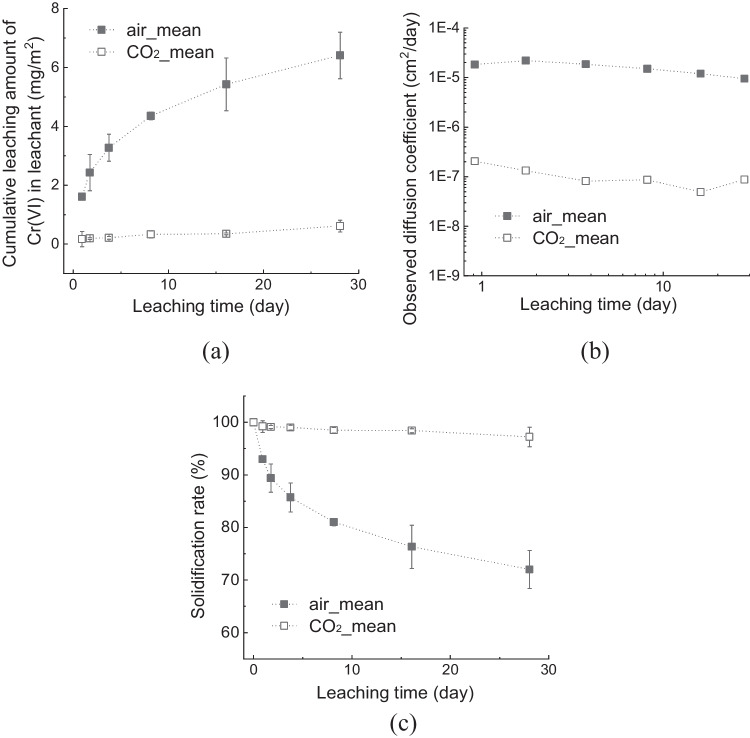
Result of tank leaching test with 28-day cured specimens (a) Cumulative leaching amount of Cr(VI), (b) Observed diffusion coefficient, *D*_*i*obs_, and (c) Solidification rate

Figure 4a shows the cumulative amount of Cr(VI) in the leachate from the cured OPC specimens. The cumulative amount of Cr(VI) in the leachate was expressed as mg per m^2^ of the specimen surface area. In the air-mixed specimens, the cumulative amount of released Cr(VI) gradually increased to 6.41 mg/m^2^ in 28 d. However, the cumulative Cr(VI) leaching from the CO_2_-mixed specimens was ten times lower (0.614 mg/m^2^) at the end of the leaching test period. Moreover, on the 1st or 2nd day of the tank leaching test, the leachate of the CO_2__cr0 samples was undetectable using a UV–visible spectrometer.

**Table 3:** 28-day cured specimens for tank leaching test

Name	No	Mass (g)	Height (cm)
	1	21.95	2.30
	2	22.16	2.35
air_cr0	3	22.14	2.35
	4	21.12	2.15
	5	21.60	2.30
	1	22.59	2.45
	2	22.60	2.45
CO_2__cr0	3	22.78	2.45
	4	21.18	2.25
	5	22.66	2.40

Figure 4b shows the logarithmic form of *D*_obs_, expressed in cm^2^ per day. The *D*_obs_ was calculated as the mean cumulative amount of Cr(VI) released during the leaching test. Similar to the two experimental conditions, the *D*_obs_ were gently declined during the applied test period. The *D*_obs_ aligned at approximately 7.66 × 10^–6^ cm^2^/day in air-mixed specimens and 1.19 × 10^–7^ cm^2^/day in CO_2_-mixed specimens. Therefore, CO_2_ mixing suppressed the mobility of Cr(VI)-containing compounds in the specimens compared with air mixing.

The solidification rates *s* of Cr(VI) in the specimens were determined using Eq. 5.

5 $$s=\frac{{C}_{0}-{C}_{t}}{{C}_{0}}\times 100,$$

where *C*_0_ is the initial leachable content of Cr(VI) in the OPC powder, and *C*_t_ is the total amount of Cr(VI) released during the test period. Figure 4c shows the solidification rate as a function of leaching time. Under the air-mixed condition, *s* dropped consistently over time immediately after the leaching test started and reached 72.04% at the end of the test period. In contrast, there was almost no significant decrease from the beginning of the leaching test under the CO_2_-mixed conditions, resulting in an *s* value of 97.22% at the end of the test period.

The mitigation of Cr(VI) release from the CO_2_-mixed samples can be attributed to the change in the cement hydrates or porosity of the cement specimens under CO_2_-mixing conditions. These results also indicate the transformation of Cr(VI)-containing cement hydrates after CO_2_ mixing.

### XRD analysis

**Fig. 5:**
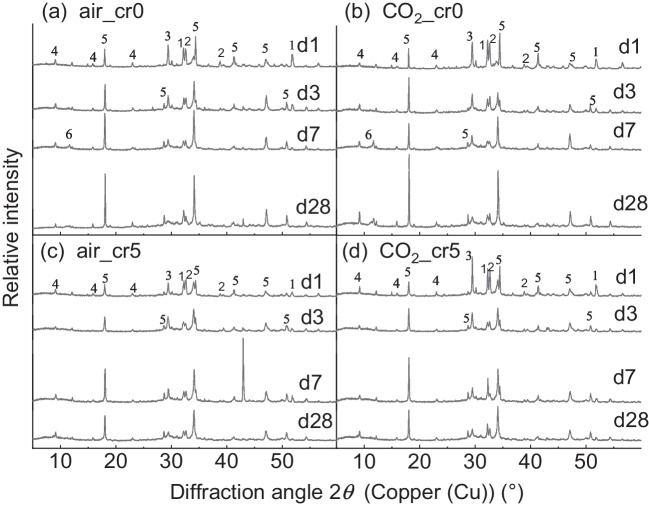
Result of XRD analysis for (a) air mixing with water, (b) CO_2_ mixing with water, (c) air mixing with Cr(VI) solution, and (d) CO_2_ mixing with Cr(VI) solution (1 = Alite (3CaO·SiO_2_), 2 = Belite (2CaO ·SiO_2_), 3 = Calcite (CaCO_3_), 4 = Ettringite (Ca_6_·Al_2_· (OH)_12_·(SO_4_)_3_·26H_2_O), 5 = Portlandite (Ca(OH)_2_), 6 = Monocarboaluminate (Ca_4_·Al_2_· (OH)_12_·CO_3_·5H_2_O))

Figure 5 shows the XRD analysis results of air and CO_2_ mixing for the samples cured for 1, 3, 7, and 28 d. Figure 5a and 5b show the results for the cement paste specimens made of water, and Fig. 5c and 5d show the results for the Cr(VI) solution. In common with all the samples, the fundamental peaks were confirmed, such as alite (3CaO·SiO_2_), belite (2CaO·SiO_2_), calcite (CaCO_3_), ettringite, or portlandite (Ca(OH)_2_).

**Fig. 6:**
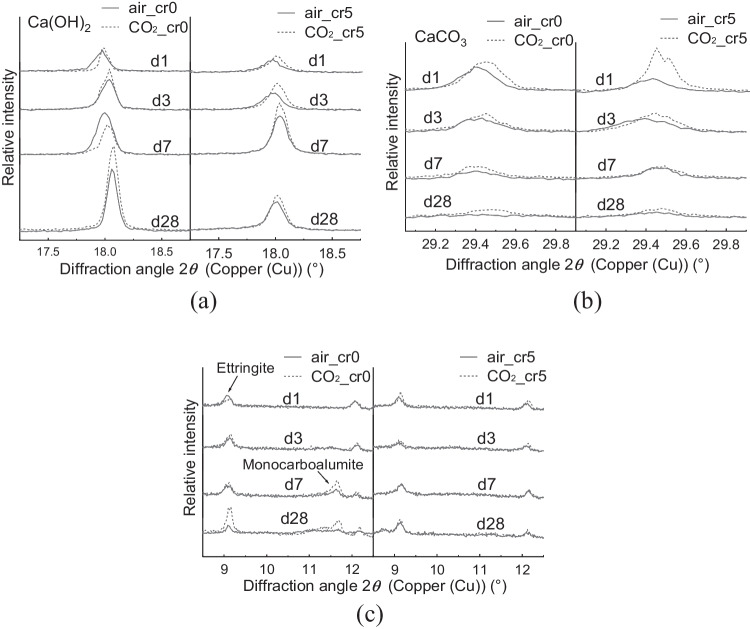
Enlarged graph of XRD analysis for (a) Ca(OH)_2_ at 17.5–18.5º, (b) CaCO_3_ at 28.0–18.5º, and (c) Monocarboaluminate at 11.0–12.5º and Ettringite at 9.0–9.5º

The Ca(OH)_2_ peak at approximately 18° is shown in Fig. 6a. The peak increased as curing proceeded in air_cr0 and CO_2__cr0. Meanwhile, the peaks for both air_cr5 and CO_2__cr5 decreased from 7 to 28 d of curing. This might be because of the lowered pH of the cement pore solution owing to Cr(VI) addition (Cau Dit Coumes et al. 2006) or the contribution of Ca(OH)_2_ for the formation of chromate hydrate, such as CaCrO_4_·2H_2_O (Bakhshi et al. 2019; Wang and Vipulanandan 2000).

The CaCO_3_ peak at approximately 29.5° is shown in Fig. 6b. Clearly, the peak of CO_2__cr5 was higher, which might be a consequence of the higher CO_2_ uptake, as shown in Fig. 3. In addition, both CO_2__cr0 and CO_2__cr5 exhibit slightly higher intensities than air_cr0 and air_cr5, implying that more CaCO_3_ is generated by CO_2_ mixing.

The monocarboaluminate (Mc, Ca_4_·Al_2_·(OH)_12_·CO_3_·5H_2_O) was observed at approximately 11.5° and ettringite was approximately 9.2° as magnified in Fig. 6c. In the case of air_cr0 and CO_2__cr0, Mc peaks were observed after 7 d of curing; in particular, CO_2__cr0 exhibited a more intense peak than air_cr0. For ettringite, CO_2__cr0 exhibited a higher peak after 28 d of curing. In contrast, neither air_cr5 nor CO_2__cr5 showed peaks of Mc. Furthermore, the ettringite peak did not change in CO_2__cr5. Instead of generating Mc or ettringite, monochromate or chromate-ettringite was assumed to be generated (Leisinger et al. 2012; Rae et al. 2022).

### TGA result

**Fig. 7:**
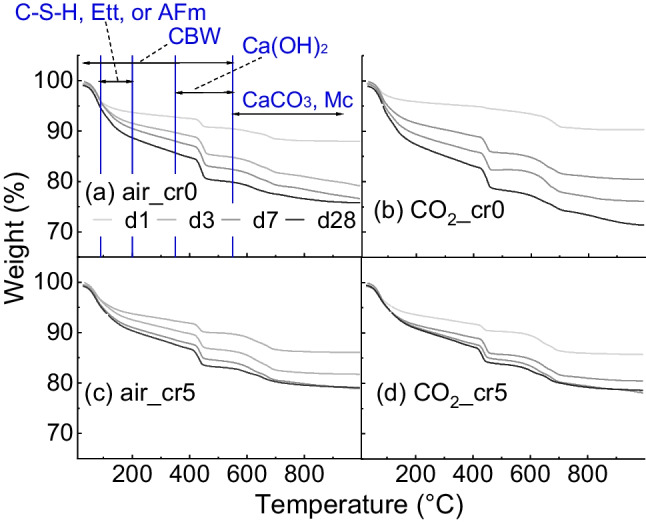
Result of TGA for (a) air mixing with water, (b) CO_2_ mixing with water, (c) air mixing with Cr(VI) solution, and (d) CO_2_ mixing with Cr(VI) solution (C-S–H = calcium silicate hydrate, Ett = ettringite, AFm = monosulfoaluminate hydrates, CBW = chemically bounded water, Mc = monocarboaluminate)

Figure 7 shows the TGA results for both the air and CO_2_ mixing samples with water and Cr(VI) solution. The weight reduction appeared at approximately 90–200 °C for the decomposition of H_2_O for cement hydrates such as calcium silicate hydrate (C-S–H), ettringite, or monosulfoaluminate hydrates (Ca_4_·Al_2_·(OH)_12_·SO_4_·6H_2_O, AFm), 350–550 °C for Ca(OH)_2_, and 550–1000 °C for CO_2_-bearing hydrate such as CaCO_3_ or Mc. It can be observed that weight reduction increased with the curing days under all experimental conditions. The weight reductions in air_cr0 and CO_2__cr0 resulted in 23.55% and 27.89% in 28-day cured samples; however, air_cr5 and CO_2__cr5 samples were 20.33% and 20.97%, respectively. CO_2_-mixing resulted in greater hydrate formation than air mixing. However, adding the Cr(VI) solution caused less hydrate generation than water mixing.

**Fig. 8:**
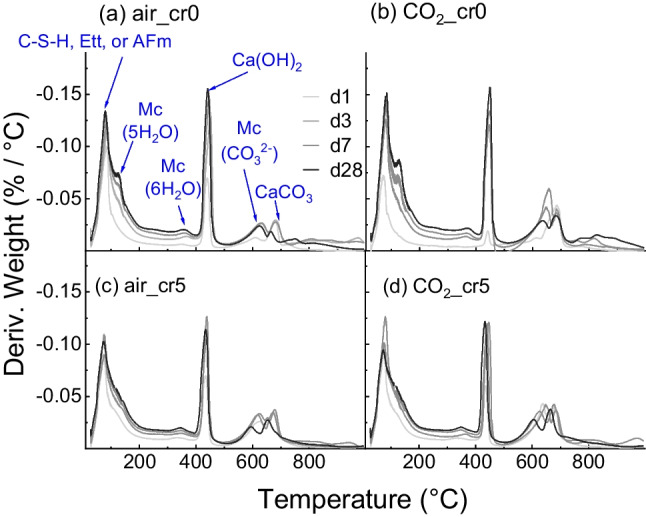
Result of DTG for (a) air mixing with water, (b) CO_2_ mixing with water, (c) air mixing with Cr(VI) solution, and (d) CO_2_ mixing with Cr(VI) solution (C-S–H = calcium silicate hydrate, Ett = ettringite, AFm = monosulfoaluminate hydrates, CBW = chemically bounded water, Mc = monocarboaluminate)

**Fig. 9:**
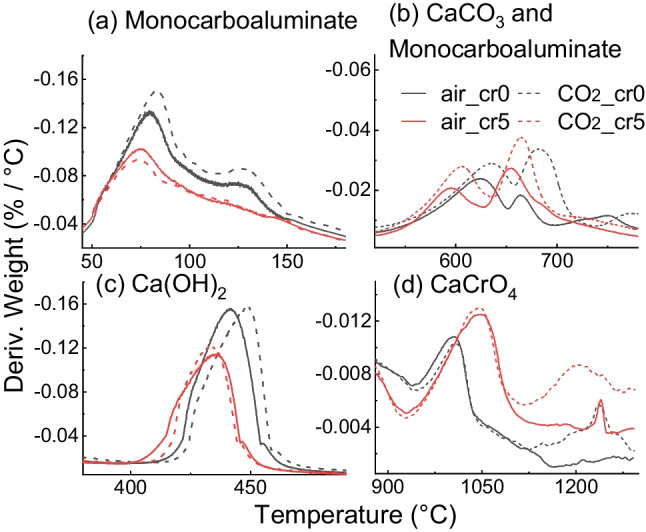
Enlarged graph of DTG analysis for (a) 5H_2_O of Monocarboaluminate (b) CaCO_3_ and CO_3_^2−^ of Mc at 500–750 °C (c) Ca(OH)_2_ at 400–500 °C and (d) CaCrO_4_ at 1000–1300 °C

Subsequently, a differential thermogravimetric analysis (DTG) was performed, as shown in Fig. 8. Peaks for C-S–H, ettringite or AFm, Ca(OH)_2_, and CaCO_3_ were mainly observed. Figure 9 shows the peaks of the 28-day cured samples and compares the amounts of each hydrate. In Fig. 9a, there is a peak at approximately 130 °C in air_cr0 and CO_2__cr0, which implies the decomposition of 5H_2_O in the interlayer of Mc and a comparatively small amount of 3H_2_O of AFm (Scrivener et al. 2016). The peak of CO_2__cr0 was comparatively higher than that of air_cr0; therefore, CO_2_ mixing increased Mc production. Meanwhile, the peaks of 5H_2_O of Mc were neither confirmed in air_cr5 nor CO_2__cr5, despite the peak at approximately 380 °C indicating the 6H_2_O of Mc regardless of the experimental conditions.

Figure 9b enlarged the neighbouring two peaks observed at approximately 500–750 °C. The peaks indicate the decomposition of CO_3_^2−^. The peak at 700 °C represents CaCO_3_ as highly crystalline, whereas the peak at 650 °C indicates Mc defined as weakly crystalline (Scrivener et al. 2016). Interestingly, the peaks for CaCO_3_ and Mc were confirmed irrespective of the mixing method and the Cr(VI) addition, although the 5H_2_O in the interlayer of Mc at 160 °C was not.

Moreover, even though the absorption amount of CO_2_ was higher in the Cr(VI) added mixing (Fig. 3), the decomposition of CO_3_^2−^ was less in CO_2__cr5 than that of CO_2__cr0; the weight reductions of CO_2__cr0 and CO_2__cr5 were 6.40% and 4.76% respectively. This can be attributed to the partial replacement of CO_3_^2−^ in Mc or CaCO_3_ was partially replaced with CrO_4_^2−^ in the cement hydrates (Ohya et al. 2012). Furthermore, because the Mc peaks existed in the CO_2__cr5 specimen at 650 °C, there is the possibility that the CO_3_^2−^ and CrO_4_^2−^ were not completely exchanged but coexisted in the cement structure. That is, a transformation of the Mc structure occurred (Ohya et al. 2012; Rae et al. 2022).

In addition, as shown in Fig. 9c, by enlarging the Ca(OH)_2_ peak, air_cr0 and CO_2__cr0 showed an increase in the peak; however, air_cr5 and CO_2__cr5 slightly decreased. This difference can be attributed to the consumption of Ca^2+^ ions during the generation of CaCrO_4_. This was also confirmed in the measurement of above 1000 °C as shown in Fig. 9d. Comparing the 28-day curing samples at 1000–1300 °C, the two peaks at approximately 1020 °C and 1200 °C were observed, representing the decomposition of CaCrO_4_ (Mao et al. 2015; Wu et al. 2020a). The water-soluble Cr(VI) is immobilised as CaCrO_4_ and deoxidised to Cr(III) above 1000 °C, as represented by Eq. 6.

6 $$4{\text{CaCrO}}_{4}\to 2\text{Ca}{\left({\text{CrO}}_{2}\right)}_{2}+3{\text{O}}_{2}+2\text{CaO}$$

The weight reductions between 1000–1300 °C were higher in CO_2_ mixing compared with air mixing; air_cr0, CO_2__cr0, air_cr5, and CO_2__cr5 decreased to 0.87%, 1.17%, 1.80%, and 2.40%, respectively. Accordingly, CO_2_ mixing appeared to affect the generation of cement hydrates, particularly Mc, CaCO_3_, Ca(OH)_2_, and CaCrO_4_. More Cr(VI) immobilisation was confirmed in CO_2_ mixing samples. The binding of Ca^2+^ and Cr(VI) or Cr(III) could be further evaluated by X-ray photoelectron spectroscopy (XPS) (Guo et al. 2024).

### FTIR analysis

**Fig. 10:**
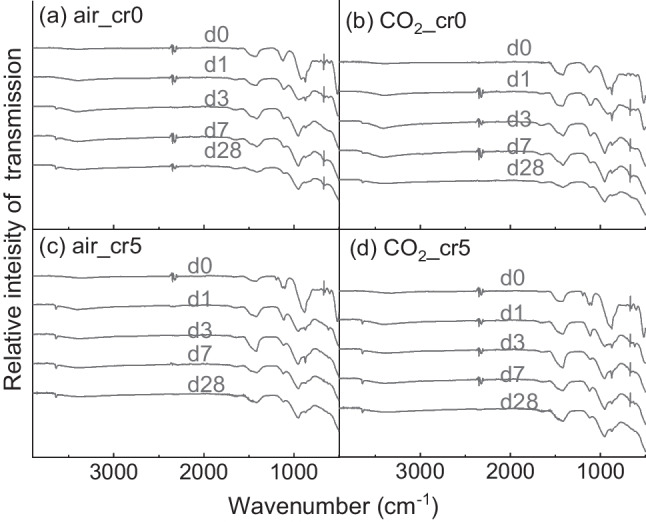
Result of FTIR for (a) air mixing with water, (b) CO_2_ mixing with water, (c) air mixing with Cr(VI) solution, and (d) CO_2_ mixing with Cr(VI) solution

The results of the FTIR analysis are shown in Fig. 10. The bands at 920 cm^−1^ indicate the Si–O vibration of silicate, which were shifted to 950 cm^−1^ as the polymerisation and generation of C-S-Has hydration progressed (Zhang and Scherer 2011). The elongations at 1125 cm^−1^ and 1100 cm^−1^ were designated as S–O vibrations, which converged to 1120 cm^−1^ as the curing progressed. The bands between 3100–3700 cm^−1^ were the result of H_2_O molecules. The C-O vibration of CaCO_3_ appeared at 1420 cm^−1^. The bands at 874 cm^−1^ were inferred from the overlapping vibrations of Cr–O and Al–OH of the ettringite.

**Fig. 11:**
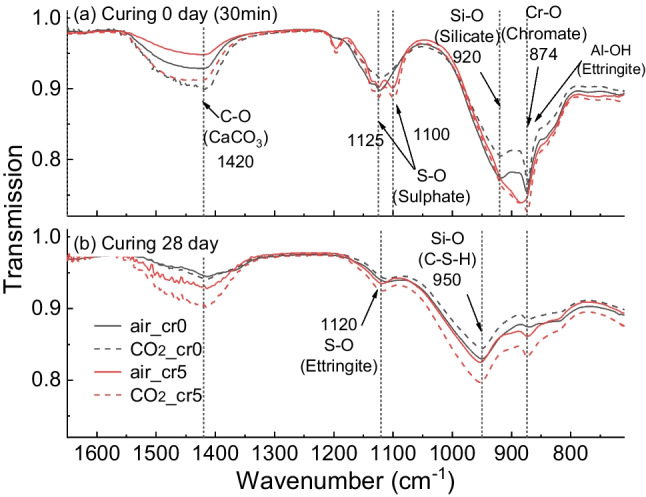
FTIR result enlarged at 800–1600 cm^−1^ at (a) immediately after the mixing (i.e., 0-day curing) and (b) 28-day curing

Figure 11 is enlarged and compares the range 800–1600 cm^−1^. In Fig. 11a, curing for 0 d implies the hydration stoppage had been performed immediately after the mixing of cement paste. In the C-O bands, CO_2_ mixing samples stretched more than air mixing. At 28-day curing, shown in Fig. 11b, the CO_2__cr5 had remained at almost the same transmission intensity as that in 0-day curing, whereas CO_2__cr0 resulted in a decrease and aligned with air_cr0. Therefore, CO_2__cr5 resulted in a significant amount of CO_3_^2−^ as the form of CaCO_3_. In addition, considering the outcomes of the XRD and TGA, it might be inferred that the reduced CO_3_^2−^ in CO_2__cr0 had become Mc or Hemicarboaluminate, whose C-O band is known as possessing other vibration wavelengths (Horgnies et al. 2013).

Furthermore, the peak at 874 cm^−1^ was higher in air_cr5 and CO_2__cr5 than in air_cr0 and CO_2__cr0 samples in 0-day curing, which seemed to be due to the addition of Cr(VI). Later, the 28-day curing represented a higher peak in CO_2__cr5 than in the other treatments. This inferred that CO_2_ mixing influenced the development of the immobilisation of CrO_4_^2−^ ions. Thus, CO_2_ mixing increased the Cr(VI) immobilisation capacity.

### Assessing the effect of in situ CO_2_ mixing on the Cr(VI) release

Based on the tank leaching test results, in situ CO_2_ mixing reduced the amount of Cr(VI) leached from the cement specimens. The specimens with in situ CO_2_ mixing resulted in 0.614 mg/m^2^ of Cr(VI) leaching, which was almost ten times less than that in the control mixing (i.e. air mixing).

In accordance with the microstructural analysis, structural changes in the cement hydrates were confirmed in the CO_2_-mixed samples. Compared to air-mixed samples, CO_2_-mixed samples with 28-day curing exhibited the following features: (1) the total weight reduction in TGA was 27.89%, which was higher than 23.55% of air-mixed samples, indicating a lower porosity in CO_2_-mixed samples, and (2) more Mc and ettringite were observed in XRD, suggesting the possibility of the generation of more monochromate or chromate-ettringite. Therefore, the lower porosity and increment in Mc and ettringite generation possibly suppressed the mobility of Cr(VI), mitigating *D*_obs_ to 1.19 × 10^–7^ cm^2^/day and leading *s* to 97% after the applied test period.

Furthermore, this study found Cr(VI)-immobilised structures in CO_2_-mixed cement paste. In CO_2_-mixed samples, (1) a relatively large amount of CrO_4_^2−^ was immobilised as CaCrO_4_; this is because a larger amount of CaCrO_4_ was formed in the TGA at temperatures higher than 1000 ºC and a higher Cr–O extension was observed in the FTIR and (2) the partial disappearance of Mc and increment of CaCrO_4_ peak were detected in TGA, suggesting the incorporation of the CrO_4_^2−^ ion to the internal layer of Mc.

This finding indicates a potential contribution to both the challenges of the CO_2_ footprint and Cr(VI)-induced environmental problems in the cement industry. While this study was performed by use of OPC with comparatively small sized specimen. Therefore, mortar or concrete experiments near the field scale and environment should be performed to evaluate the actual CO_2_ absorption and pollutant immobilization performance. Further, the fresh cement paste should be elucidated in more detail in terms of pH changes and valance changes of chromium. These measurements can be beneficial for further discussion in Cr-immobilisation structures in cement. Moreover, considering the contamination of groundwater or the soil environment, further evaluation is desirable under the conditions examined in field environments, such as pH change owing to acid rain and structural changes owing to the potential mixing with industrial by-products (e.g. fly ash or ground granulated blast furnace slag). Combined with an experimental database of heavy metal leaching and the generation of cement hydrates, it is feasible to establish a monitoring system for underground heavy metal pollution.

## Conclusion

This study evaluated the effect of a laboratory-scale in situ CO_2_ mixing method on the leachability of hexavalent chromium (Cr(VI)) from cement specimens. Tank leaching tests revealed that in situ CO_2_ mixing reduced Cr(VI) leaching from the cement specimens. The CO_2_-mixed samples showed only 0.614 mg/m^2^ of Cr(VI) leaching, almost ten times less than the air-mixed samples. Microstructural analysis confirmed structural changes in the CO_2_-mixed cement hydrates. These samples exhibited lower porosity, with a 27.89% weight reduction in thermogravimetric analysis (TGA) and higher monocarboaluminate and ettringite generation. These changes effectively lowered Cr(VI) mobility, reducing the observed diffusion coefficient (*D*_obs_) to 1.19 × 10^–7^ cm^2^/day and achieving 97% of solidification rate (*s*) under the applied test period. This study also identified Cr(VI)-immobilised structures in the CO_2_-mixed cement, including CaCrO_4_ formation and CrO_4_^2−^ incorporation into Mc in the CO_2_-mixed samples. These findings demonstrate that CCUS technology, which has been the main focus of attention in recent years, is beneficial for reducing CO_2_ emissions and, from an environmental perspective, controlling the leaching of hazardous substances.

## Data Availability

The datasets are available from the corresponding author upon reasonable request.
